# COVID-19: a multi-organ perspective

**DOI:** 10.3389/fcimb.2024.1425547

**Published:** 2024-10-18

**Authors:** Fabiana Amaral Guarienti, João Ismael Budelon Gonçalves, Júlia Budelon Gonçalves, Fernando Antônio Costa Xavier, Daniel Marinowic, Denise Cantarelli Machado

**Affiliations:** ^1^ Graduate Program in Biomedical Gerontology, School of Medicine, Pontifical Catholic University of Rio Grande do Sul, Porto Alegre, RS, Brazil; ^2^ Brain Institute of Rio Grande do Sul (BraIns), Pontifical Catholic University of Rio Grande do Sul (PUCRS), Porto Alegre, RS, Brazil

**Keywords:** SARS-CoV-2, Covid-19, hyperinflammation, systemic effects, clinical outcomes

## Abstract

In this mini review, we explore the complex network of inflammatory reactions incited by SARS-CoV-2 infection, which extends its reach well beyond the respiratory domain to influence various organ systems. Synthesizing existing literature, it elucidates how the hyperinflammation observed in COVID-19 patients affects multiple organ systems leading to physiological impairments that can persist over long after the resolution of infection. By exploring the systemic manifestations of this inflammatory cascade, from acute respiratory distress syndrome (ARDS) to renal impairment and neurological sequelae, the review highlights the profound interplay between inflammation and organ dysfunction. By synthesizing recent research and clinical observations, this mini review aims to provide an overview of the systemic interactions and complications associated with COVID-19, underscoring the need for an integrated approach to treatment and management. Understanding these systemic effects is crucial for improving patient outcomes and preparing for future public health challenges.

## 1 Introduction

COVID-19, caused by the severe acute respiratory syndrome coronavirus 2 (SARS-CoV-2), has rapidly evolved from a regional health crisis into a global pandemic, affecting millions worldwide and exerting unprecedented strain on public health systems (Chams et al., 2020; Sohrabi et al., 2020; Sharma et al., 2021). Initially characterized as a respiratory illness, extensive research has since uncovered that its impact extends far beyond the lungs, affecting multiple organ systems and leading to a myriad of clinical manifestations (Ramos-Casals et al., 2021; Golzardi et al., 2024). This systemic involvement is largely mediated by an intense inflammatory response, which not only targets the respiratory system but also has profound effects on cardiovascular, hepatic, digestive, renal, reproductive, and neurological functions (Elrobaa and New, 2021; Ramos-Casals et al., 2021; Saed Aldien et al., 2022).

The pathophysiology of COVID-19 is complex and multifaceted, involving direct viral injury and a dysregulated immune response (Khalil et al., 2022; Merad et al., 2022). The multi-organ damage observed in COVID-19 patients can be attributed in part to the wide distribution of SARS-CoV-2 receptors across various tissues and organs. The primary receptor for SARS-CoV-2 is angiotensin-converting enzyme 2 (ACE2), which facilitates viral entry into host cells (Lan et al., 2020; Shang et al., 2020a; Shang et al., 2020b). ACE2 is abundantly expressed not only in the respiratory tract but also in the cardiovascular system, gastrointestinal tract, kidneys, liver, and central nervous system (Hikmet et al., 2020; Chen et al., 2021). This widespread expression pattern explains the diverse range of symptoms and complications observed in COVID-19 patients. In addition to ACE2, other receptors and co-receptors, such as neuropilin-1 (NRP1) and the transmembrane serine protease 2 (TMPRSS2), are also widely expressed across tissues and play significant roles in facilitating SARS-CoV-2 entry and propagation within host cells (Jiang et al., 2022; Karki and Kanneganti, 2022; Michalski et al., 2022; Collado-Lledó et al., 2024). The co-expression of these receptors in various tissues enhances the virus’s ability to infect multiple organ systems, further contributing to the systemic effects of the disease.

**Figure 1:**
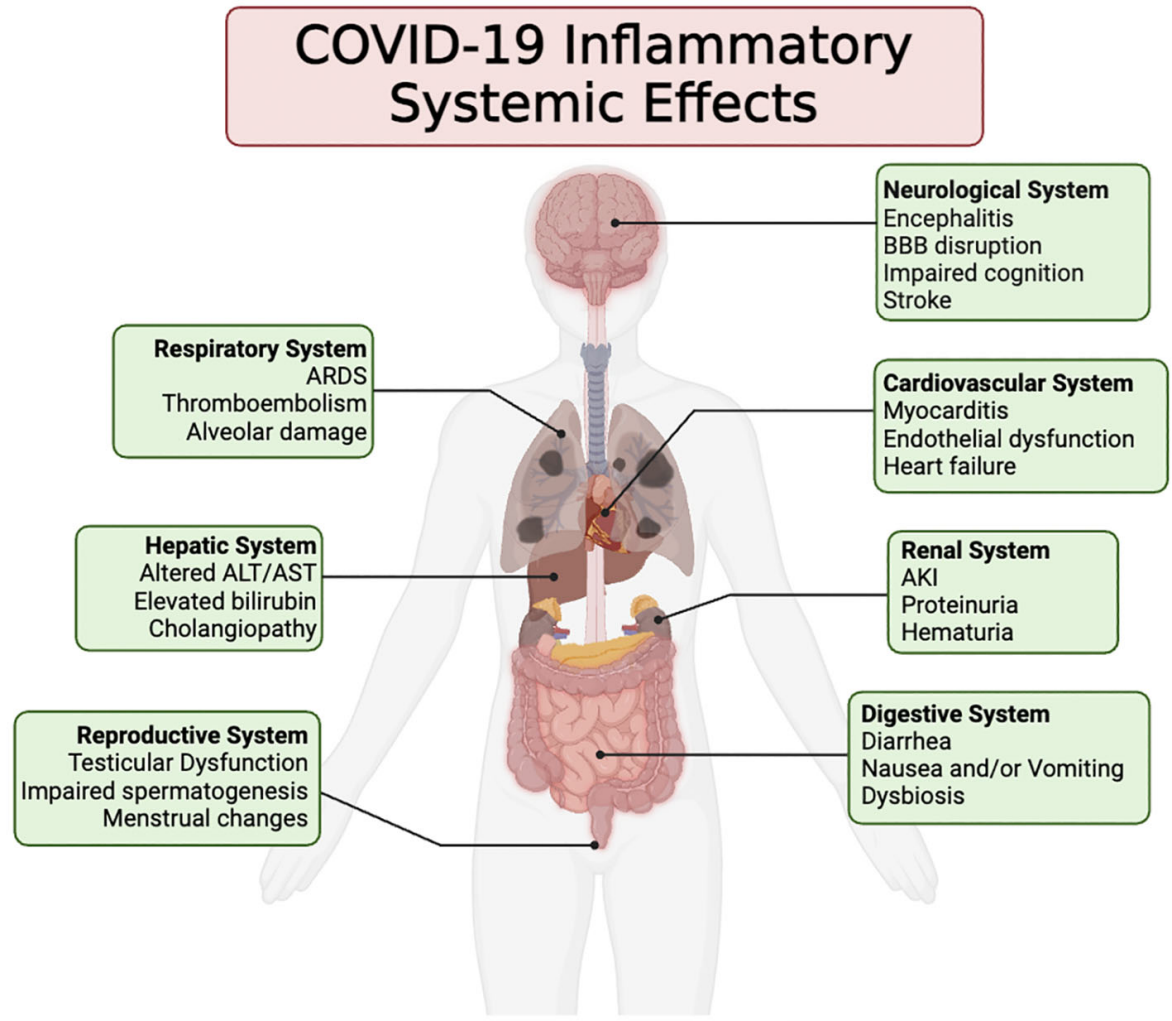
This illustration portrays the wide array of clinical symptoms linked to the inflammatory reaction induced by COVID-19 across different organ systems. These symptoms may arise either directly from the inflammatory response or indirectly as a result of the inflammation incited following the infection and demise of the host’s cells by SARS-CoV-2.

This viral spread triggers a cascade of immune reactions, including the release of pro-inflammatory cytokines, which can lead to a state known as cytokine storm (Jiang et al., 2022; Karki and Kanneganti, 2022). This hyperinflammatory response is responsible for much of the severe morbidity and mortality associated with the disease, contributing to conditions ranging from acute respiratory distress syndrome (ARDS) to multi-organ failure (**Figure 1**) (Mishra et al., 2020; Michalski et al., 2022; Collado-Lledó et al., 2024).

This mini-review aims to provide an overview of the systemic effects of the inflammatory response in COVID-19 patients, highlighting the key mechanisms and manifestations across various organ systems. By synthesizing data from various research studies and clinical observations, our aim is to emphasize the interplay between organ systems in the context of COVID-19, emphasizing the importance of a comprehensive approach in addressing this complex disease. Recognizing these systemic connections is essential not just for the optimal clinical care of COVID-19 patients but also for anticipating and addressing future complexities in global health dynamics.

## 2 Respiratory system

The respiratory system serves as the primary target for SARS-CoV-2 (Van Slambrouck et al., 2023). The virus can infect various cells, including nasal and bronchial epithelial cells, goblet cells, and ciliated cells (Zou et al., 2020; Ahn et al., 2021; Ravindra et al., 2021; Gamage et al., 2022; Osan et al., 2022; Otter et al., 2023). Recent studies have highlighted the specific impact of SARS-CoV-2 on airway motile cilia. SARS-CoV-2 preferentially replicates in multiciliated cells and induces their dedifferentiation in a reconstructed human bronchial epithelium model, leading to a rapid loss of motile cilia and impaired mucociliary clearance (Robinot et al., 2021). This phenomenon was also observed in SARS-CoV-2-infected hamsters, where a loss of motile cilia in the trachea was documented (Ahn et al., 2021). The early attachment of SARS-CoV-2 into ciliated cells facilitates viral entry, which can be blocked by depleting cilia or accelerated by depleting mucins (Wu et al., 2023). The virus causes significant microvilli rearrangement and expansion in epithelial cells, activating critical kinases (PAK1, PAK4, SLK) for viral spread. Inhibiting these kinases can block viral spread without affecting initial binding, indicating potential therapeutic targets. SARS-CoV-2 exits via microvilli, forming viral chains that facilitate dissemination, while inhibitors disrupting microvilli severely impair viral exit (Ravindra et al., 2021).

**Figure 2:**
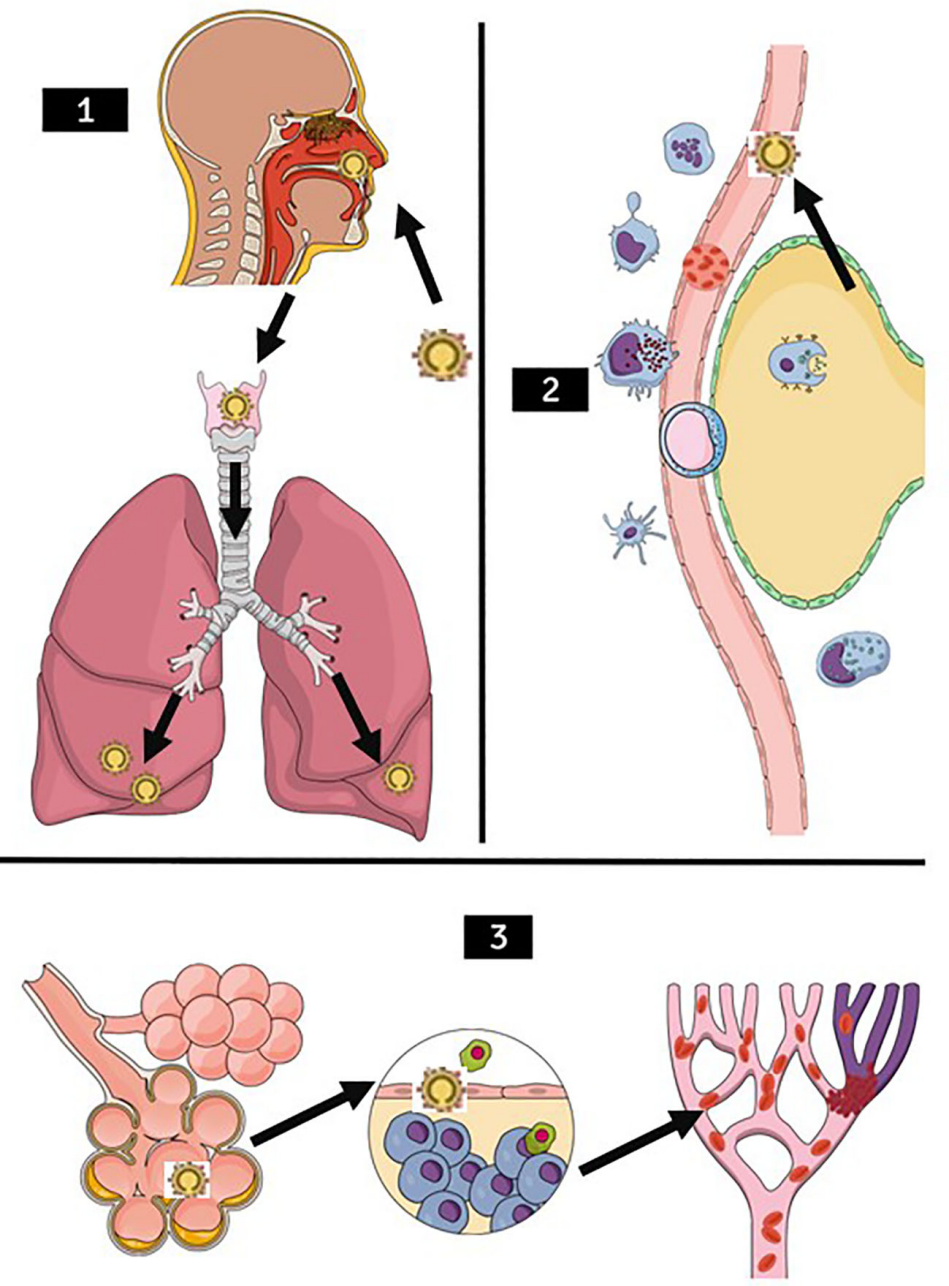
Stages of lung damage through SARS-CoV-2 illustration. (1) SARS-CoV-2 infection pathway (arrows) through the upper airways, migrating mainly to the lower lung lobes. (2) Recruitment of immune cells such as macrophages, neutrophils, lymphocytes, antigen-presenting cells, natural killer cells in the presence of SARS-CoV-2 (arrow). (3) Worsening of the clinical condition, with blood hypercoagulability occurring, first generating pulmonary edema, in the second figure the defense cells entering the alveoli causing an inflammatory reaction, with a higher probability to decrease oxygen saturation and also, one of the very common outcomes accompanied by D- dimers increased, the Pulmonary Thromboembolism presentation.

Following viral attachment and entry, a cascade of inflammatory responses is triggered, involving the release of pro-inflammatory cytokines and recruitment of immune cells (Blanco-Melo et al., 2020; Cambridge Institute of Therapeutic Immunology and Infectious Disease-National Institute of Health Research (CITIID-NIHR) COVID-19 BioResource Collaboration et al., 2021; Iwasaki et al., 2021) (**Figure 2**). This leads to local tissue damage and exacerbation of inflammation within the respiratory tract. COVID-19 often manifests as pneumonia characterized by bilateral ground-glass opacities and consolidation on chest imaging (Jain et al., 2021; Hemraj et al., 2022). Histopathological studies of affected lungs reveal diffuse alveolar damage, hyaline membrane formation, and infiltration of inflammatory cells, indicative of acute respiratory distress syndrome (ARDS) (Borczuk et al., 2020; Xu et al., 2020; Batah and Fabro, 2021; Caramaschi et al., 2021). The alveolar damage compromises gas exchange, leading to hypoxemia and respiratory failure, necessitating mechanical ventilation in severe cases (Jin et al., 2018; Calkovska et al., 2021; Dushianthan et al., 2023).

COVID-19 is associated with a heightened risk of thromboembolic events, including pulmonary embolism and microvascular thrombosis within the pulmonary vasculature (Riyahi et al., 2021; Overton et al., 2022; Wada et al., 2023). Endothelial dysfunction, hypercoagulability, and systemic inflammation contribute to the pathogenesis of COVID-19-associated coagulopathy, further exacerbating respiratory compromise and organ dysfunction (Jin et al., 2020; Xu SW. et al., 2023; Valencia et al., 2024).

Respiratory issues emerge as a prevalent phenotype among individuals experiencing long COVID, with studies indicating a twofold increase in occurrence compared to the general population (Bull-Otterson et al., 2022). Among the array of respiratory symptoms, shortness of breath and cough stand out as the most prevalent, persisting for at least seven months in a notable proportion of long COVID patients (Davis et al., 2021).

Additionally, various imaging investigations involving non-hospitalized long COVID patients have unveiled pulmonary irregularities such as air trapping and altered lung perfusion (Yu et al., 2022). A recent study analyzing a cohort of more than 112,000 individuals revealed a continuous increase in respiratory disorders among COVID-19 survivors, including asthma, bronchiectasis, COPD, ILD, PVD, and lung cancer, with severity of acute COVID-19 correlating with heightened risk. Over a 24-month follow-up, risks of asthma and bronchiectasis continued to rise, underscoring the importance of long-term monitoring and follow-up care for these patients (Meng et al., 2024).

## 3 Cardiovascular system

**Figure 3:**
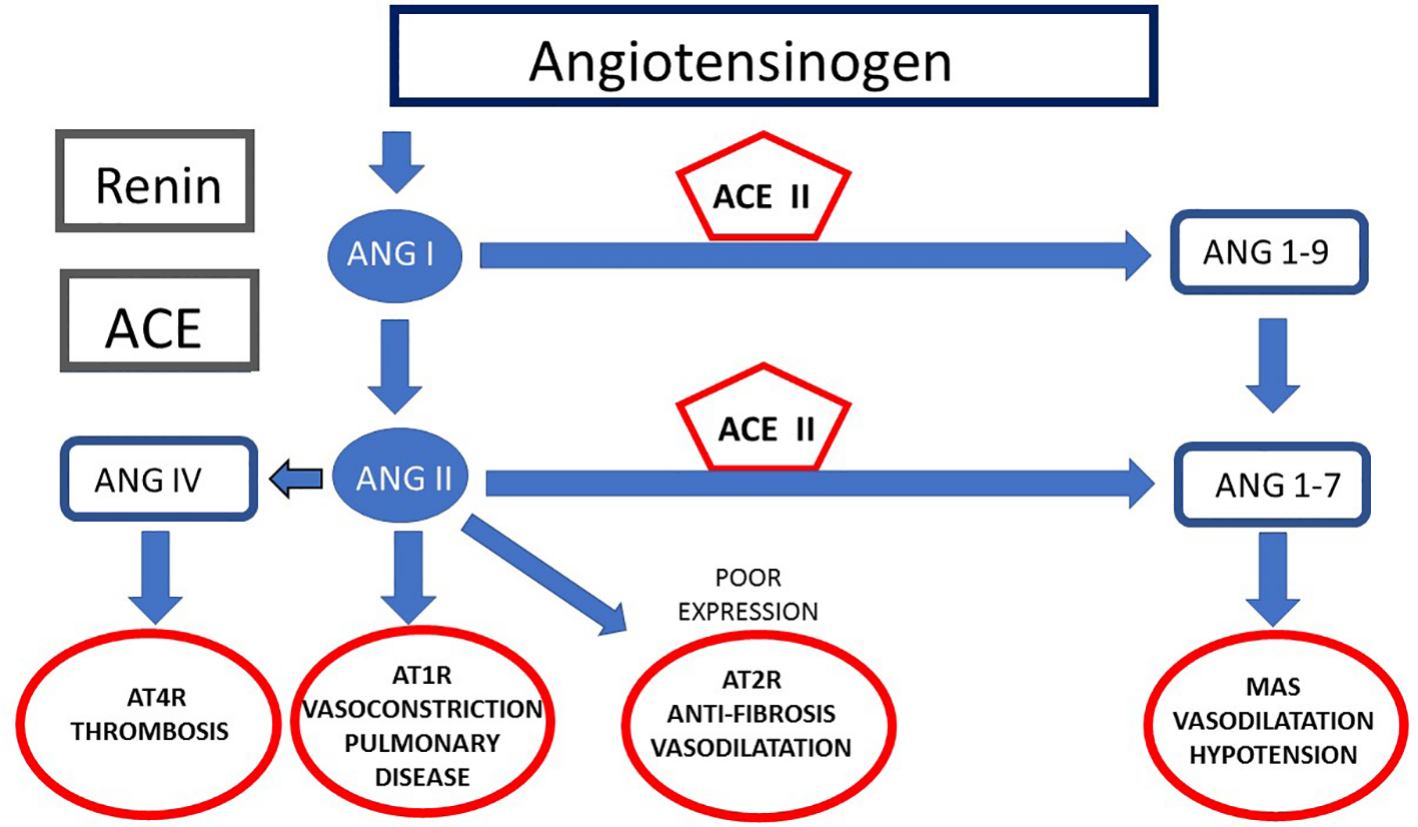
Functioning of Angiotensinogen. This illustration demonstrates the Renin-Angiotensin System cascade, highlighting the conversion of Angiotensinogen into Angiotensin I and II, and the vasoconstrictive action of Angiotensin II. The role of ACE2 in converting Ang II into Ang 1-7, demonstrates one cycle benefit to human body, in which promotes vasodilation. The ACE2 interaction with coronavirus during COVID-19 are also represented. During the disease the ACE2 is working as the main virus receptor, reducing its work in this cycle making diminished Ang 1-9 or Ang 1-7 production. The different receptors AT1R, AT2R, AT4R, and MasR are indicated, with their functions in blood pressure regulation, fibrosis, and thrombosis. During the disease, AT4R and AT1R are the main receptors working in this cycle.

The cardiovascular implications of COVID-19 can be attributed to several mechanisms. Firstly, direct viral invasion of cardiomyocytes (CMs) has been reported (Bojkova et al., 2020; Bailey et al., 2021; Marchiano et al., 2021; Yang et al., 2021). Additionally, both *in vitro* studies using human induced pluripotent stem cells (hiPSCs) and isolated adult CMs, as well as *in vivo* experiments with animal models, have shown that CMs are susceptible to SARS-CoV-2 infection (Bojkova et al., 2020; Marchiano et al., 2021; Yang et al., 2021). Single-cell expression analysis has shown that ACE2 receptors are highly expressed in CMs from healthy heart tissues (Liu et al., 2020). Interestingly, the expression levels of ACE2 in the adult human heart are higher than those in the lungs (**Figure 3**).

Apart from directly infecting cells, SARS-CoV-2 can also impair cardiovascular function by targeting endothelial cells and pericytes within blood vessels (Puelles et al., 2020; Varga et al., 2020; Liu et al., 2021; Brumback et al., 2023). This interaction can lead to endothelial dysfunction, a pivotal factor in the pathogenesis of atherosclerosis and thrombosis. Recent research indicates that the virus has the potential to infect coronary arteries (Eberhardt et al., 2023). This infection may result in inflammation and destabilization of plaque buildup, increasing the risk of plaque rupture and subsequent heart attack (Eberhardt et al., 2023).

The clinical manifestations of cardiovascular involvement in COVID-19 patients are diverse and can range from mild symptoms to severe, life-threatening conditions. These include myocarditis, arrhythmias, pericardial effusion, acute coronary syndromes, myocardial injury, myocardial infarction, new-onset or worsening heart failure, arterial and venous thromboembolism, cardiogenic shock, and cardiac arrest (Huang et al., 2020; Šikić et al., 2021; Daugherty et al., 2021; Xie et al., 2022).

Endothelial dysfunction, characterized by impaired vascular homeostasis and increased pro-thrombotic activity, plays a pivotal role in COVID-19-related cardiovascular complications (Bonaventura et al., 2021). Inflammatory mediators disrupt endothelial function, leading to vasoconstriction, microvascular thrombosis, and atherosclerosis (Cybulsky and Gimbrone, 1991; Puhlmann et al., 2005; Hansson and Hermansson, 2011; Zhan and Rockey, 2011; Gimbrone and García-Cardeña, 2016; Tawakol et al., 2017). COVID-19-associated coagulopathy, characterized by elevated D-dimer levels and disseminated intravascular coagulation, further exacerbates thrombotic events, including pulmonary embolism and myocardial infarction (Cui et al., 2020; Tang et al., 2020; Zhou et al., 2020).

COVID-19-induced inflammation can directly affect the heart, leading to myocardial injury characterized by elevated cardiac biomarkers (e.g., troponin, B-type natriuretic peptide) and myocardial dysfunction (Puntmann et al., 2020; Shi et al., 2020). Myocarditis, an inflammatory condition of the myocardium, has been reported in COVID-19 patients, presenting as chest pain, arrhythmias, and heart failure (Craver et al., 2020; Shafi et al., 2020). Additionally, SARS-CoV-2 infection may exacerbate pre-existing cardiovascular conditions, such as hypertension, coronary artery disease, and heart failure, through systemic inflammation and hemodynamic stress.

Among individuals hospitalized due to COVID-19, hypertension and diabetes emerge as the most common comorbidities. In a study comprising 5,700 patients, hypertension was found to be the predominant comorbidity, affecting 56.6% of the cohort (The Task Force for the management of COVID-19 of the European Society of Cardiology, 2022). Recent investigations consistently indicate that COVID-19 patients with hypertension face an elevated risk of mortality compared to those without hypertension (Abdi et al., 2022).

An examination of over 150,000 individuals from the US Department of Veterans Affairs one year following SARS-CoV-2 infection, revealed a notable increase in the risk of various cardiovascular diseases. This increased risk encompassed conditions such as heart failure, dysrhythmias, and stroke, irrespective of the initial severity of the COVID-19 presentation (Xie et al., 2022).

Therefore, COVID-19 exerts profound effects on the cardiovascular system, ranging from acute cardiac injury and thrombotic complications to chronic cardiovascular sequelae. A comprehensive understanding of the cardiovascular manifestations of COVID-19 is crucial for guiding clinical management and optimizing cardiovascular outcomes in affected individuals.

## 4 Hepatic system

The liver is susceptible to COVID-19 through several pathways. The virus can directly infect hepatocytes and cholangiocytes, facilitated by the ACE2 receptor expressed in these cells, leading to cellular damage and liver dysfunction (Zhao et al., 2020; Barnes, 2022; Wanner et al., 2022). Moreover, the systemic inflammatory response triggered by COVID-19, characterized by elevated levels of pro-inflammatory cytokines (e.g., IL-6, TNF-a), can exacerbate liver injury through immune-mediated mechanisms and microvascular thrombosis (Sanyaolu et al., 2023). Hypoxic injury from severe respiratory distress and hypoxemia, common in critical cases, can also compromise the liver’s oxygen supply, leading to further hepatic damage (Huang et al., 2021).

Liver involvement in COVID-19 is primarily indicated by abnormal liver function tests, observed in a significant proportion of patients. The most common hepatic abnormalities include mild to moderate elevations in aminotransferases (AST and ALT), suggesting hepatocellular injury (Guo et al., 2020; Huang et al., 2020). Some patients also exhibit elevated bilirubin levels, indicative of liver dysfunction or biliary injury (Said et al., 2023). Recent investigations into COVID-19 have revealed varying incidences of liver injury, ranging from 14.8% to 53% (Fan et al., 2020; Huang et al., 2020).

COVID-19 can also lead to coagulopathy, reflected in altered coagulation profiles with prolonged prothrombin time, especially in severe cases (Leentjens et al., 2021; Ragnoli et al., 2023). The COVID-19 induced coagulopathy, in turn, may affect the liver through the formation of microthrombi in the liver’s microcirculation. These microthrombi obstruct blood flow, causing ischemia and oxygen deprivation (Zhao et al., 2021). Severe cases of COVID-19 are marked by a hyperinflammatory response that can lead to multi-organ failure. Research indicates that elevated levels of inflammatory cytokines in the blood are closely linked to signs of liver dysfunction in patients suffering from COVID-19 (Zhu et al., 2021).

There has been a growing number of reports on post-COVID-19 cholangiopathy among adults. This condition encompasses prolonged cholestasis and secondary sclerosing cholangitis (Bütikofer et al., 2021; Faruqui et al., 2021; Meersseman et al., 2021; Roth et al., 2021). In general, the long-term hepatic consequences of COVID-19 remain unclear. However, there is concern that severe disease and pre-existing liver conditions might lead to worsened liver function or accelerated progression of chronic liver disease.

Additionally, liver damage could also be related to the presence of co-infections with other viruses such as dengue. Studies indicate that co-infections of COVID-19 and dengue can exacerbate liver injury, leading to a worse prognosis (Verduyn et al., 2020; Reyes-Ruiz et al., 2021).

## 5 Digestive system

SARS-CoV-2 directly infects the gastrointestinal tract, leading to a spectrum of symptoms including diarrhea, nausea/vomiting, abdominal pain, anorexia, loss of taste, and elevated liver enzymes (Yusuf et al., 2021; Xu E. et al., 2023). These manifestations arise from various mechanisms, including mucosal barrier disruption, inflammatory responses, and alterations in the composition of the gut microbiota (Zang et al., 2020; Zuo et al., 2020; Livanos et al., 2021; Cheng et al., 2022). Dysbiosis of the gut microbiota, characterized by alterations in microbial composition and diversity, further exacerbates inflammation and intestinal barrier dysfunction, creating a favorable environment for viral replication and systemic immune activation (Di Vincenzo et al., 2024).

Many COVID-19 patients experience gastrointestinal symptoms alongside typical respiratory symptoms such as fever and cough, with varying prevalence rates ranging from 3% to 79% (Cholankeril et al., 2020; Ferm et al., 2020; Pan et al., 2020; Redd et al., 2020). Anorexia is the most common GI symptom, followed by diarrhea and nausea/vomiting, while abdominal pain is less frequently reported. In pediatric patients, gastrointestinal symptoms are more prevalent compared to adults. A recent meta-analysis found increased rates of nausea/vomiting (19.7%) and abdominal pain (20.3%), although diarrhea prevalence (19.08%) did not exhibit a significant difference (Bolia et al., 2021). Furthermore, SARS-CoV-2 RNA has been detected in the feces of newborns and mothers, as well as in breast milk and the placenta, suggesting potential routes of vertical transmission and additional considerations for maternal and neonatal health (Hinojosa-Velasco et al., 2020; Kilic et al., 2021). This finding underscores the need for further research to understand the implications of these transmission routes and their impact on gastrointestinal and overall health in both mothers and infants.

A recent large-scale retrospective study showed that individuals previously infected with COVID-19 have higher risks of various digestive diseases, including gastrointestinal dysfunction, peptic ulcer disease, gastroesophageal reflux disease (GERD), gallbladder disease, severe liver disease, non-alcoholic liver disease, and pancreatic disease (Ma et al., 2024). The severity of the acute phase of COVID-19 correlates with increased GERD risk. Even after a year, GERD and gastrointestinal dysfunction continue to pose risks. Reinfection with SARS-CoV-2 further increases the risk of pancreatic diseases (Pan et al., 2020). A comprehensive understanding of the gastrointestinal manifestations and hepatic consequences of COVID-19 is crucial for guiding clinical practice and optimizing patient care in affected individuals.

## 6 Renal system

First reports have indicated a potential high incidence of acute kidney injury (AKI) in COVID-19 patients, speculated to reach up to 25% (Birkelo et al., 2021; Chan et al., 2021). Autopsy findings have provided evidence of viral affinity for the renal system. Additionally, a significant proportion of COVID-19 patients, around 60% of 147 individuals, reportedly experienced proteinuria, with 48% also presenting with hematuria (Li Z. et al., 2020). Laboratory results further confirmed renal involvement, with elevated blood urea nitrogen (BUN) and creatinine levels observed in patients (Kilic et al., 2021).

The pathophysiology of COVID-19-associated AKI is multifactorial and may involve direct viral cytopathic effects, systemic inflammation, cytokine-mediated renal injury, microvascular thrombosis, and hemodynamic instability (Golmai et al., 2020; Miller and Brealey, 2020; Legrand et al., 2021; De Las Mercedes Noriega et al., 2023). SARS-CoV-2 can directly infect renal tubular epithelial cells via the angiotensin-converting enzyme 2 (ACE2) receptor (Carrau et al., 2023; Radovic et al., 2023). leading to tubular dysfunction, interstitial inflammation, and acute tubular necrosis (Braun et al., 2020; Radovic et al., 2023). The activation of the renin-angiotensin-aldosterone system and complement system further amplifies renal injury and inflammation in COVID-19 patients (Sullivan et al., 2022).

A multicenter study performed using patients from multiple UK hospitals reported that among over 85,000 patients, approximately 2.6% required acute kidney replacement therapy (KRT). Of those with available data, around 31.5% exhibited biochemical evidence of acute kidney injury (AKI), with varying severity levels. Chronic kidney disease (CKD), male sex, and Black race were identified as primary risk factors for both KRT and biochemical AKI. The risk of mortality within 28 days increased with the severity of AKI (Legrand et al., 2021).

Severe COVID-19-associated AKI necessitates renal replacement therapy, encompassing modalities such as intermittent hemodialysis, continuous renal replacement therapy, and peritoneal dialysis (Doher et al., 2021; Shemies et al., 2022). Moreover, emerging evidence indicates that COVID-19 survivors may face long-lasting renal issues like CKD, proteinuria, and renal fibrosis (Bowe et al., 2021; Huart et al., 2021)Further longitudinal studies are needed to fully understand how COVID-19-related renal problems affect kidney function and outcomes over time.

## 7 Reproductive system

COVID-19 negative effects on the reproductive system are frequently reported during long COVID, yet extensive research documenting the full extent of these impacts and the sex-specific pathophysiology remains scarce.

Regarding the male reproductive health, recent findings indicate that severe cases of COVID-19 might lead to testicular damage, potentially caused by the direct invasion of testicular cells by the SARS-CoV-2 virus or through the infection of immune cells, followed by excessive immune activation (Costa et al., 2023). One study showed that macrophages are one of the main SARS-CoV-2 lodging sites in the testes of severe COVID-19 patients (Carrau et al., 2023). Furthermore, elevated levels of activated mast cells were also present in testicular tissue, regarded as a promoter of inflammation in the tissue (Carrau et al., 2023). The immune response triggered by SARS-CoV-2 infection could also play a role in testicular dysfunction, posing a potential risk to reproductive health (Li H. et al., 2020; Li X. et al., 2020; Yang et al., 2020; Duarte-Neto et al., 2022; Li et al., 2022). This damage could present as impaired spermatogenesis, decreased testosterone production, and changes in semen quality, which might have implications for male fertility and reproductive outcomes (Garrouch et al., 2023; Meng et al., 2024). Additionally, studies have shown that COVID-19 can negatively affect sperm parameters, including sperm concentration, motility, and morphology, and may also be associated with erectile dysfunction in young individuals post-COVID-19 (Jiménez-López et al., 2023; GamalEl Din et al., 2024).

Menstrual changes are more prevalent among women and individuals who menstruate experiencing long COVID compared to those without a history of COVID and those who had COVID-19 but not long COVID (Medina-Perucha et al., 2022). Patients have reported that menstruation and the premenstrual week can trigger relapses of long COVID symptoms (Davis et al., 2021). Furthermore, decreased ovarian reserve and disorders in reproductive endocrine function have been noted in individuals affected by COVID-19 (Ding et al., 2021). The underlying mechanisms of COVID-19-associated menstrual changes remain poorly understood but may involve systemic inflammation, stress, and immune dysregulation.

Pregnant women infected with COVID-19 are at increased risk of developing severe disease and pregnancy complications, including preterm birth, preeclampsia, and maternal-fetal transmission of the virus (Li X. et al., 2020; Yang et al., 2020; Duarte-Neto et al., 2022). SARS-CoV-2 may cross the placental barrier and infect fetal tissues, potentially leading to fetal developmental abnormalities, intrauterine growth restriction, and neonatal morbidity and mortality (Naidu et al., 2022). Close monitoring and management of pregnant women with COVID-19 are essential to optimize maternal and neonatal outcomes.

## 8 Neurological and cognitive systems

Emerging evidence suggests that COVID-19 can have significant neurological and cognitive consequences, both in the acute phase of infection and in the long-term (Crivelli et al., 2022; Hartung et al., 2022; Xu et al., 2022; Rothstein, 2023). A scoping review by Wenting et al. examined 85 articles on the neurological manifestations of COVID-19 and found that they can range from mild symptoms like loss of taste/smell, dizziness, and headaches to more severe complications like ischemic stroke and encephalitis (Wenting et al., 2020).

SARS-CoV-2 gains entry into the CNS via the olfactory nerve or through hematogenous spread, facilitated by the expression of the ACE2 receptor in neuronal and glial cells (Meinhardt et al., 2021; Patrì et al., 2021; Krasemann et al., 2022; Albornoz et al., 2023). Meinhardt et al. measured SARS-CoV-2 RNA load in various regions, including the oropharyngeal and nasopharyngeal areas, and CNS areas such as the olfactory bulb, medulla, and cerebellum. Viral RNA was found in the CNS, particularly in the olfactory bulb and medulla, in about one-third of the samples. Notably, higher CNS viral RNA loads correlated with shorter disease duration (Meinhardt et al., 2021).

The analysis of post-mortem COVID-19 patients identified increases in activated monocytes/macrophages and perivascular macrophages in the CNS, suggesting infiltration of pro-inflammatory monocytes. Additionally, activated microglia were found in the brain parenchyma, indicating a neuroinflammatory response (Matschke et al., 2020). Microglial activation was observed in response to S protein, potentially triggering neuroinflammation (Schwabenland et al., 2021; Olajide et al., 2022; Fontes-Dantas et al., 2023). It was also reported that T cells in the CNS of COVID-19 patients were activated, expressing markers of exhaustion, cytotoxic granules, and proliferation (Schwabenland et al., 2021). Additionally, the dysregulated hyperinflammation triggered by COVID-19 involves the release of pro-inflammatory cytokines, IL-6, TNF-α, and IL-1β, which contribute to neuroinflammation, blood-brain barrier disruption, neuronal damage and impaired cognitive function (Normandin et al., 2021; Pilotto et al., 2021; Bonetto et al., 2022; Fernández-Castañeda et al., 2022; Soung et al., 2022).

Studies have reported that COVID-19-induced inflammation can contribute to a range of neurological and cognitive complications, including impaired cognition, memory decline, and the development of neuropsychiatric disorders, such as depression and anxiety (Woo et al., 2020; Kupcova et al., 2023; Loftis et al., 2023; Hampshire et al., 2024). The long-term implications of these neurological and cognitive effects are still being investigated, with some COVID-19 survivors experiencing persistent neurological symptoms even after recovery (García-Sánchez et al., 2022; Krishnan et al., 2022). Furthermore, the presence of pre-existing neurological or cognitive conditions, such as Alzheimer’s disease or Parkinson’s disease, may exacerbate the neurological and cognitive consequences of COVID-19 (Martín-Jiménez et al., 2020). The interplay between COVID-19-induced inflammation and the underlying pathophysiology of these neurological disorders requires further exploration.

Encephalitis is a severe neurological complication observed in pediatric COVID-19 patients. Cases of SARS-CoV-2-associated encephalitis in children have been reported, highlighting the potential for the virus to induce significant neuroinflammatory responses in younger populations. These cases often present with symptoms such as seizures, altered mental status, and focal neurological deficits (Sánchez-Morales et al., 2021; Valderas et al., 2022; Reyes-Ruiz et al., 2023).

COVID-19 has also been associated with a range of ophthalmic manifestations. These include conjunctivitis, dry eye syndrome, and more severe conditions such as retinopathy and optic neuritis (Kumar et al., 2021; Al-Namaeh, 2022). Studies have reported these symptoms in various populations, underscoring the importance of recognizing and addressing ocular involvement in COVID-19 patients (Kumar et al., 2021; Al-Namaeh, 2022; Shaikh et al., 2022; Hernández-Reyes et al., 2023). The ophthalmic manifestations of COVID-19 may result from direct viral invasion, immune-mediated damage, or secondary effects of systemic inflammation.

## 9 Conclusion and future perspectives

The COVID-19 pandemic has underscored the extensive systemic impacts of viral infections like SARS-CoV-2, which extend far beyond respiratory symptoms to affect multiple organ systems. Initially, the focus was primarily on pulmonary symptoms, but it soon became clear that the disease had a broader impact. The subsequent emergence of severe complications—including liver dysfunction, elevated D-dimer levels, rapid onset of metabolic acidosis, cardiopulmonary edema, renal failure, respiratory insufficiency, and the need for mechanical ventilation—highlighted the profound and multifaceted nature of the disease. This rapidly escalated to significant ICU admissions, coma, and a high mortality rate among previously healthy individuals, presenting a major challenge to global health systems.

A critical factor in the severity of COVID-19 is the dysregulated immune response, which leads to a cytokine storm. Although cytokines are intended to protect the host from infection, their excessive and uncontrolled release exacerbates the condition. Additionally, alterations in ACE2 expression play a crucial role. ACE2 is involved in various physiological processes, including the metabolism of bradykinin and the regulation of the renin-angiotensin system.

Bradykinin, a vasodilator, is normally metabolized by ACE into an inactive form, Bradykinin 1-5. When ACE activity is disrupted, bradykinin levels can increase, contributing to vasoconstriction and exacerbating the inflammatory response. ACE also influences neurokinins, which are involved in pain transmission, emotional regulation, and immune response modulation. In the lungs, Angiotensin I (ANG I) is converted to Angiotensin II (ANG II), which binds to the AT1 receptor, causing vasoconstriction, hypertension, and promoting inflammation. ANG II can also be converted to angiotensin IV (ANG IV), which is associated with thrombosis. ACE2 counteracts these effects by converting ANG II into Angiotensin (1–7), which binds to the Mas receptor, leading to vasodilation and hypotension. Thus, ACE2 reduces the renin-angiotensin system’s effects and mitigates the vasoconstriction, fibrosis, and hypertrophy induced by SARS-CoV-2. ACE2 receptors are widely expressed in various tissues, with significant expression in the lungs and intestines, explaining the prevalent gastrointestinal infections during the pandemic and contributing to patient deterioration.

These interactions not only lead to acute systemic complications, such as multi-organ failure and vascular aggravation, but may also have long-term health implications for survivors. Addressing hyperinflammation, disturbances in ACE2 function, and alterations in immune mechanisms is crucial for both acute management and long-term care of COVID-19 patients.

Future research should aim to elucidate the mechanisms behind COVID-19-induced hyperinflammation to develop targeted therapies. Enhancing vaccines and antiviral strategies to address new variants is essential. Moreover, the global health community must strengthen public health infrastructure and enhance international cooperation to better prepare for future pandemics.
